# Emerging Human Metapneumovirus Gene Duplication Variants in Patients with Severe Acute Respiratory Infection, China, 2017–2019

**DOI:** 10.3201/eid2701.201043

**Published:** 2021-01

**Authors:** Zhibo Xie, Jin Xu, Yunhui Ren, Aili Cui, Huiling Wang, Jinhua Song, Qiang Zhang, Manli Hu, Wenbo Xu, Yan Zhang

**Affiliations:** National Institute for Viral Disease Control and Prevention, Beijing, China (Z. Xie, A. Cui, H. Wang, J. Song, Q. Zhang, M. Hu, W. Xu, Y. Zhang);; Henan Provincial Center for Disease Control and Prevention, Zhengzhou, China (J. Xu);; Luohe Prefectural Center for Disease Control and Prevention, Luohe, China (Y. Ren)

**Keywords:** acute respiratory tract infections, China, genetic variations, human metapneumovirus, Metapneumovirus, Pneumoviridae, severe acute respiratory infection, viruses, respiratory infection

## Abstract

We detected human metapneumovirus (HMPV) in 72 (7.1%) of 1,021 patients hospitalized with severe acute respiratory infection in Luohe, China, during 2017–2019. We detected HMPV most frequently in young children and less often in adults. HMPV genotype A2c variants 111 nt and 180 nt duplications predominated, demonstrating their continuing geographic spread.

Human metapneumovirus (HMPV; family *Pneumoviridae*, genus *Metapneumovirus*) is a major cause of acute respiratory tract infections, especially in children and elderly persons (*1*,*2*). Its genome is ≈13.2 kb, containing 8 genes encoding 9 proteins. The G gene, around 654–867 nt acids sequence length, is the most variable nucleotide sequence in the whole genome of HMPV and has been widely used to study HMPV genetic variation (*3*–*5*). HMPV has 1 serotype with 2 subgroups A and B, further divided into 5 genotypes, including A1, A2a, A2b, B1, and B2, based mainly on variations in the G gene (*4*,*6*,*7*). Recently, unique HMPV variants possessing a 180 nt duplication (nt-dup) in the G gene, first reported in Spain, and a 111 nt-dup in the G gene, first reported Japan (*5*,*8*,*9*), followed by Croatia and Guangdong, China (*10*,*11*). These variants were clustered in the A2c lineage of the phylogenetic tree (*11*). In this study, we investigated the prevalence of HMPV associated with patients with severe acute respiratory infection (SARI) and identified genetic variations in the G gene of HMPV in Luohe, in Henan Province, China, during 2017–2019. 

## The Study

Luohe is a city of 2.8 million people in Henan Province, northern China. In this study, we collected throat swab specimens and clinical data from 1,021 patients with SARI admitted to Luohe Central Hospital during October 2017–June 2019. Patients were 1 month–95 (median 3) years of age; most (76.7%) of the SARI cases involved children <5 years old. All swab specimens were tested by multiplex real-time reverse transcription PCR assay using a nucleic acid diagnostic kit (Kinghawk, http://www.kinghawk828.com), which identifies HMPV, influenza types A and B, human respiratory syncytial virus (RSV), human coronaviruses, human rhinovirus and enterovirus, human adenovirus, human parainfluenza viruses, and human bocavirus.

**Figure 1:**
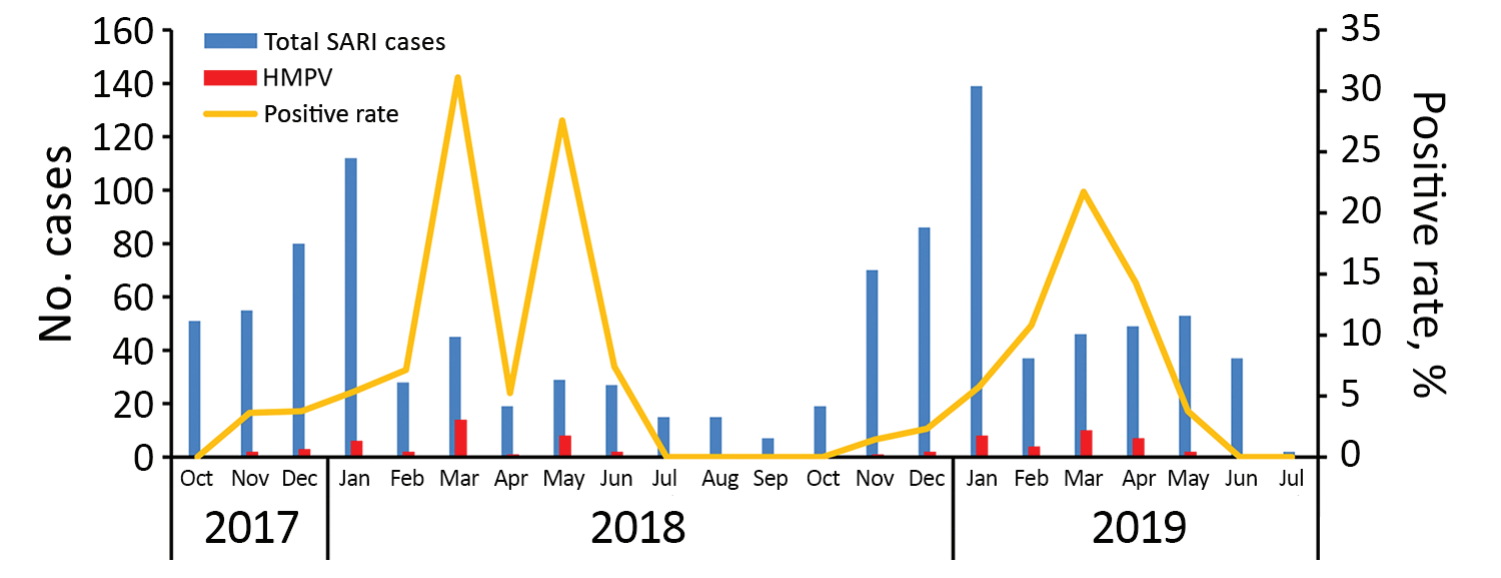
Distribution by month of SARI cases and HMPV-positive rates in Luohe, China, 2017–2019. HMPV, human metapneumovirus; SARI, severe acute respiratory infection.

Overall, 83.2% (849/1021) of SARI patients were positive for >1 respiratory viruses; human adenovirus was the predominant virus identified, at 22% (225/1021). HMPV was identified in 7.1% (72/1021) of SARI patients, consistent with other studies that identified HMPV in 6%–12% of SARI cases (*12*,*13*). The proportion of HMPV-positive patients with co-detected respiratory viruses was 63.9% (46/72). The rates of HMPV positivity decreased gradually with age, from 9.1% in those <2 years old to 0.9% in adults (Table 1). The epidemic season for HMPV lasted from November through May or June, with the peak number of cases occurring in March and May in 2018 and March in 2019 (Figure 1). Most (98.5%) HMPV-positive patients in this study exhibited cough or dyspnea and were diagnosed with bronchopneumonia (74.6%). No clear differences in clinical signs and symptoms were apparent among the patients infected with duplication variants compared with other HMPV viruses.

**Table 1:** Age composition of the 1,021 SARI patients with HMPV-positive samples from patients with SARI admitted to Luohe Central Hospital, Luohe, China, during October 2017–June 2019*

Age, y	SARI	HMPV-positive	HMPV-positive, by age, total, %
<2	385	35 (9.1)	48.6
2–4	351	26 (7.4)	36.1
5–17	171	10 (5.9)	13.9
18–95	113	1 (0.9)	1.4
Total	1,021	72 (7.1)	100.0

*HMPV, human metapneumovirus; SARI, severe acute respiratory infection.

**Figure 2:**
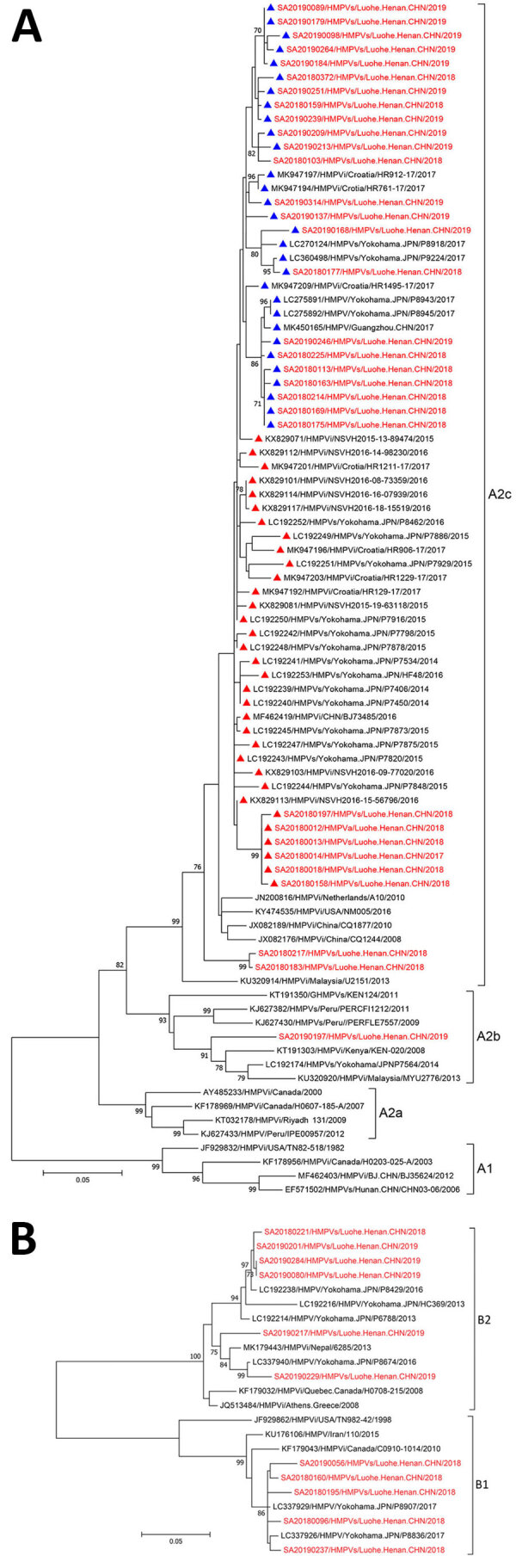
Phylogenetic tree generated by maximum-likelihood method of HMPV G gene sequences (red) from patients with SARI admitted to Luohe Central Hospital, Luohe, China, during October 2017–June 2019, and reference sequences (black). A) Phylogenetic tree for the representative viruses of subgroup A (A1, A2a, A2b and A2c), including 54 representative G gene sequences of HMPV from GenBank and 32 from this study. The strains with 111 nt duplication are indicated by blue triangles; strains with 180 nt duplication are indicated by red triangles. 0.05 scale bar represents 5% genetic distances for G genes (0.05 substitutions per site). B) Phylogenetic tree for the representative viruses of the B1 and B2 genotypes, including 12 representative entire coding regions of HPMV G gene sequences from GenBank and 11 from this study. The numbers at the branch nodes indicate the statistical significance, calculated with 1,000-replication bootstrapping. The nomenclature of HMPV in this study includes the following information: the source of sequences (“s” after HMPV indicates a specimen source of sequences; “i” indicates isolates), location where the viruses were detected (city, province, and country), and collection year. The GenBank accession number was added before the nomenclature of each sequence. 0.05 scale bar represents 5% genetic distances for G genes (0.05 substitutions per site). HMPV, human metapneumovirus.

Forty-three entire coding-region sequences of the G gene were obtained from 72 HMPV-positive samples, as described elsewhere (*3*,*5*,*6*) (Table 2). The nucleotide sequences generated in this study were submitted to GenBank (accession nos. MN944056–97). We selected 86 subgroup A sequences (54 from GenBank and 32 from this study) and 23 subgroup B sequences (12 from GenBank and 11 from this study) to construct the phylogenetic tree by maximum-likelihood method (Figure 2). The HMPV sequences obtained in this study were clustered into 4 genotypes, including A2c, A2b, B1, and B2 (Table 2; Figure 2). Twenty-eight out of 31 A2c sequences were grouped together in 1 distinct cluster with the duplication variants, mainly detected in Spain, Japan, Croatia, and Guangzhou, China. In the sequence alignment comparison analysis, we identified 22 viruses containing 111 nt-dup variants and 6 viruses contained 180 nt-dup variants in the G gene. The 111 nt-dup variants were detected in Luohe in 2018 and continued to spread in 2019, while 180 nt-dup variants were detected during 2017 and 2018 (Table 2). The 111 nt-dup variants were separated from the 180 nt-dup variants in the phylogenetic tree, which indicates that the 2 duplication variants evolved by independent patterns in different evolutionary lineages (Figure 2, panel A). 

**Table 2:** Distribution of HMPV positive samples and genotypes by year among patients with SARI admitted to Luohe Central Hospital, Luohe, China, during October 2017–June 2019*

Year (months)	HMPV-positive samples	Total sequences obtained	A2b	A2c			B1	B2
				Non-dup	111 nt-dup	180 nt-dup		
2017 (Oct–Dec)	5	1	0	0	0	1	0	0
2018 (Jan–Dec)	36	21	0	3	9	5	3	1
2019 (Jan–Jun)	31	21	1	0	13	0	2	5
Total	72	43	1	3	22	6	5	6
*dup, duplication; HMPV, human metapneumovirus; SARI, severe acute respiratory infection.								

The twenty-two 111 nt-dup variants circulating in Luohe during 2018–2019 were closely related to the duplication-variant viruses identified in Japan, Croatia, and Guangdong, China, in 2017, with nucleotide identity of 95.7% to 100%. Six 180 nt-dup variants circulating in Luohe during 2017–2018 were clustered in 1 small cluster with high nucleotide identity at 99.1% and 100% and closely related to the duplication viruses identified in Japan, Croatia, Spain, and Beijing, China, during 2014–2017 (Figure 2, panel A). This finding indicates that the 111 nt-dup and 180 nt-dup variants have already spread and might spread extensively throughout the world.

## Discussion

Our findings show that 7.1% of SARI patients in the Luohe study tested positive for HMPV during 2017–2019. The rate of HMPV-positivity decreased gradually with patient age. Among the HMPV patients, 63.9% were co-infected with other respiratory viruses. Four different genotypes were cocirculating, including A2b, A2c, B1, and B2. We identified most of the A2c viruses as duplication variants, including the 111 nt-dup and 180 nt-dup variants, and found that these 2 variants were the predominant viruses circulating in Luohe during 2017–2019. 

The 111 nt-dup and 180 nt-dup variants have also been detected in different regions of the world, including in Spain, Japan, Croatia, and Guangzhou, China, in recent years (*5*,*8*–*11*). Similar duplications in the G gene have been reported in HRSV, another member of the family *Pneumoviridae*. The HRSV duplication variants of both BA9 and ON1 have rapidly spread globally, becoming the predominant viruses in many countries for many years (*14*,*15*). These findings suggest that the emerging HMPV 111 nt-dup and 180 nt-dup variants might also become predominant viruses throughout the world. 

There were some limitations in this study. The epidemic seasons were not covered for the entire 3 years, and only 1 hospital was involved in this study. Continuous surveillance will be required to determine whether these novel HMPV 111 nt-dup and 180 nt-dup variants will persist as predominant viruses and have a wider geographic distribution in the future.

Although no difference in clinical symptoms was observed between the patients infected with HMPV duplication variants and those with non–duplication-variant viruses in this study, the increased transmission frequency of the duplication variants suggests a role for duplication in the G gene in potentially expanding its transmission. Therefore, further study is needed to clarify if and how the duplications result in an evolutionary advantage for HMPV. 
